# Factors contributing to the decision to perform a cesarean section in Labrador retrievers

**DOI:** 10.1186/s12917-018-1381-8

**Published:** 2018-02-27

**Authors:** Gaudenz Dolf, Claude Gaillard, Jane Russenberger, Lou Moseley, Claude Schelling

**Affiliations:** 1 Institute of Genetics, Vetsuisse Faculty, University of Berne, Bremgartenstrasse 109a, 3001 Berne, Switzerland; 2 Guiding Eyes for the Blind, 611 Granite Springs Road, Yorktown Heights, NY 10598 USA; 3 Clinic of Reproductive Medicine, Vetsuisse Faculty, University of Zurich, Winterthurerstrasse 260, 8057 Zurich, Switzerland

**Keywords:** Cesarean section, Assisted delivery, Labrador retriever, Risk factors

## Abstract

**Background:**

In the past 10 years, the frequency of unplanned cesarean sections in the Labrador Retriever breeding colony at Guiding Eyes for the Blind stayed around 10% (range 5% to 28%). To reduce the number of cesarean sections, factors influencing the occurrence of a cesarean section need to be known. The goal of this study was to identify factors that contribute to the decision to perform a cesarean section.

**Results:**

Of the 688 Labrador Retriever litters whelped between 2003 and 2016, 667 litters had sufficient data and remained in the analysis. The target trait was ordinal with the three levels “normal whelping”, “assisted whelping” and “cesarean section”. A general ordinal logistic regression approach was used to analyze the data. Model selection with possible predictors resulted in a final model including weight of the dam, the weight of the heaviest puppy of a litter, the number of fetuses malpositioned and the quality of uterine contractions. Weight and size of a litter, parity, maternal inbreeding coefficient, whelping season, dam and sire were dropped from the model because they were not significant. The risk of a cesarean section was influenced by the combination of the weight of the dam and the weight of the heaviest puppy in the litter, as well as by the number of malpositioned fetuses and the quality of the contractions. Larger puppies increased the risk of cesarean section especially when the dam had a lighter weight. For dams weighing 23.6 kg and 32.8 kg the predicted probability of a cesarean section was low, with 0.06 and 0.02, respectively, when the heaviest puppy in a litter was light (0.42 kg), contractions were normal and no fetus was malpositioned. However, the probability of a cesarean section was much higher, ranging from 0.24 to 0.08, when the heaviest puppy in a litter was heavy (0.66 kg).

**Conclusions:**

Means to reduce the cesarean section frequency in this Labrador Retriever breeding colony should include genetic selection for ideal puppy weight. In addition, dams with an adult body weight substantially below average should not be selected as breeders in this colony.

**Electronic supplementary material:**

The online version of this article (10.1186/s12917-018-1381-8) contains supplementary material, which is available to authorized users.

## Background

A cesarean section (c-section) is the surgical intervention applied when a dam with dystocia fails to respond to the medical treatment or fetal distress is evident [1]. The term dystocia is used to describe a difficult birth or the failure of a normal vaginal delivery. In dogs, dystocia is a frequently encountered complication during parturition and for roughly 60% of these cases the decision to perform a c-section is taken [2]. Although health surveillance is an important epidemiological tool to improve canine health and welfare, the collection of disease information for dogs is still problematic [3]. Incident rates for dystocia and c-sections have been reported for the general dog population as well as for specific breeds [2, 4], but as they are based on insurance data or questionnaires they may not be representative. Based on the observation in humans that dystocia runs in families a genetic background has been suggested [5, 6]. Knock-out mice implicated several genes to be involved in dystocia [7], but the relevance of these models for dystocia in humans could not be shown [8, 9].

Maternal and fetal factors are known to cause dystocia often resulting in a c-section. The most common cause of maternal dystocia is uterine inertia whereas malpositioned fetuses are the most common cause of fetal dystocia [10]. In some dog breeds, particularities of the pelvic anatomy of the dam or the facial skeleton of a puppy may result in a predisposition for obstructive dystocia [4, 11]. Bergström and coworkers [2] identified the breed, age of the dam, and the geographical region as possible risk factors for a dam to be in need of a c-section. In addition, the veterinarian and the owner seem to have a rather high influence on the decision of a surgical intervention.

In the past ten years, the frequency of c-sections in a breeding colony of Labrador Retrievers at Guiding Eyes for the Blind never dropped markedly under 10% and even reached a high of 28% in 2012.

The aim of the present study was to identify factors, other than uterine inertia and malpositioned fetuses that have an influence on the occurrence of a c-section in the Labrador Retriever breeding colony at Guiding Eyes for the Blind and could be improved by breeding and/or adapting management.

## Methods

Guiding Eyes for the Blind is a non-for profit organization that breeds and trains mostly Labrador Retrievers (LR) and a few German Shepherd guide dogs to serve people who are blind or have visual impairment. Dams receive a complete physical examination at the start of each estrus cycle and the decision to mate is made based on receiving medical clearance and the need for puppies. The dam is tested for *Brucella canis* using a rapid slide agglutination test at the beginning of each heat where a mating is planned. Males are tested for antibodies against *Brucella canis* every 6 months. On average 65 LR matings are realized per year resulting in 61 whelpings with an average litter size of 7.9 and a litter size at weaning of 7.2. Stillborn puppies and neonatal losses account for 65% and 35% of the puppy losses, respectively.

The timing of matings is based on determining the peak fertile window of the dam through progesterone level testing. The “initial rise” (IR) generally occurs when the serum progesterone level rises to between 1.5 and 2.0 ng/ml (Antech Diagnostics – Fountain Valley, CA 92708 and in-house Tosoh 360 – Tosoh Bioscience – King of Prussia, PA 19406 progesterone assays) and typically coincides with the release of luteinizing hormone. Matings occur most often on days 3 or 4 and 5 or 6 post IR. Estrus is also monitored through vaginoscopy, vaginal cytology and receptivity.

After mating, dams are returned to their foster homes from the start of diestrus until 3 to 5 days prior to their due date which is based on 65 days post IR. An ultrasound to verify pregnancy and approximate the litter size is performed at approximately 33 days post IR.

Dams are admitted to the Guiding Eyes whelping kennel approximately 3 days prior to their due date, which allows sufficient time to acclimate to the kennel environment, and housed in a private, specially equipped whelping suite. Experienced staff monitor for prepartum changes such as nesting, changes in food intake and most notably the drop in rectal temperature below 37.2 degrees Celsius. Individual dam progesterone levels are monitored daily during the 2 to 3 days prior to the anticipated whelp date. If progesterone levels are 2 ng/ml, whelps are typically within 24–36 h; if progesterone levels are 1 ng/ml, whelps are typically within 18–24 h; if progesterone levels are below 1 ng/ml, early stages of whelp have either begun or will begin within 18 h.

Normal parturition is monitored via video monitors by staff in a nearby room with occasional visits to the whelping suite to provide needed elimination walks, food, water or support for the puppies. The decision points for assisted whelping are guided by an established protocol. A c-section will be performed if labor fails to initiate by day 66 from the progesterone IR date measured during estrus or within 24 to 36 h after the progesterone level drops below 2 ng/ml. If the dam has exhibited visible contractions without delivering a puppy the protocols depend on the presence of a fetus in the vaginal canal, strength of contractions and fetal heart rates.

In cases of obstructive dystocia where a fetus is palpated in the vaginal canal and the dam is exhibiting tail arching contractions for 30 min without delivering a puppy a vaginal examination is performed to check for malposition and/or structural maternal abnormalities. Attempts are made to reposition the malpositioned fetus through digital manipulation and/or manually deliver the puppy. The staff veterinarian is called in for a c-section if a puppy is not delivered within 20 min.

If a fetus is palpated upon vaginal examination, however tail arching contractions are very weak or absent, fetal heart rates are measured by ultrasound twice, 10 min apart. If fetal heart rates are 150 beats per minute or slower, the staff veterinarian is called in to provide support which often results in a c-section. If fetal heart rates are above 160 beats per minute, staff will feather the dorsal vagina, often resulting in stronger and/or more consistent contractions which move the fetus within reach to manually deliver the puppy. If a puppy is not delivered, fetal heart rates are monitored and if they are above 160 beats per minute, intermittent periods of allowing the dam to deliver the puppy, feathering the dorsal vagina and monitoring heart rates continue, and the veterinarian is consulted. If heart rates drop below 150 beats per minute, a c-section is performed.

In cases where no tail arching contractions have occurred for a period of two hours since a puppy was delivered and the dam is sleeping or resting the dam is taken for a short exercise walk and fed a small meal. A vaginal examination is performed to check for a fetus in the vaginal canal and fetal heart rates are measured. If fetal heart rates drop below 150 beats per minute, the staff veterinarian is called in and a c-section is performed. If fetal heart rates are at least 170 beats per minute, the vaginal examination is repeated 30 min later along with feathering. If no contractions result, the staff person handling the whelp obtains approval to administer 2 units of oxytocin (Henry Schein Animal Health – Dublin, OH 43017) frequently administered subcutaneously and occasionally by intramuscular injection along with 23% calcium solution (Vedco – Saint Joseph, MO 64507) administered subcutaneously at the dosage of 0.5 ml per 4.5 kg body weight. If within 30 min a puppy is not delivered and/or no contractions resulted from feathering, oxytocin and calcium are repeated provided fetal heart rates are at least 170 beats per minute. If within an additional 30 min a puppy is still not delivered and/or fetal heart rates drop below 150 beats per minute, the staff veterinarian is called in and a c-section is performed.

Statistical analyses were carried out using Stata/SE 14.1 (StataCorp, 4905 Lakeway Drive, College Station, Texas 77,845, USA). The initial data set comprised 688 litters of LR from 2003 to 2016 by 256 different dams and 150 sires. The target trait, ease of whelping (EOW), was ordinal with the three levels “normal whelping”, “assisted whelping without c-section” and “c-section with or without prior assistance”. Correlation coefficients between possible predictors were calculated to avoid the inclusion of highly correlated predictors. Possible predictors were the parity ranging from 1 to 6, the number of malpositioned fetuses (defined as birth position not either head first and front legs forward or rear first and rear legs extended at time of delivery), ranging from 0 to 3 or more, as well as that number squared, the quality of contractions coded 0 if normal and 1 if poor (poor was coded when medical treatment fails to improve contraction strength sufficiently to deliver the puppy), as an indicator for uterine inertia, the whelping season with 4 levels (January to March, April to June, July to September and October to December), and the inbreeding coefficient of the dam. Dam age was not included as a variable because dams are bred on a regular basis so that the age at a given parity would not differ greatly among dams. Most parities and c-sections are in dams 4 years of age and younger. The identity of the dam and the identity of the sire were evaluated as random effects. In addition, two groups of variables were considered as predictors. The first group comprised variables that describe the body condition of the dam, including the adult weight in kg, the height at withers in cm, the body mass index in kg/m^2^, and the weight (kg) to height (cm) ratio. The second group comprised variables describing the litter, including the litter size ranging from 2 to 13, the litter weight in kg, the average puppy weight in kg, the standard deviation of the puppy weights in kg, and the variance of the puppy weights in kg^2^, and the weight of the heaviest puppy in kg.

The ordinal target trait EOW was analyzed with ordered logistic regression. The different steps of the model development are described in Additional file 1.

## Results

Dropping 13 observations because c-sections were planned, 7 observations because of missing data and a single observation with parity 7 left 667 litters in the analyses. With the body mass index, height at withers or weight to height ratio in the model an additional 203 observations were lost due to missing values for the height of the dams, leaving 464 observations in the analyses. Details of the descriptive statistics of all variables are presented in Additional file 2.

*P*-values, df, Akaike’s information criterion and the Bayesian information criterion for the full and the final model are given in Table 1.

**Table 1:** Comparison of the full model and the final model for EOW

Variable	Full model	Final model
Adult weight of the dam	0.001	0.003
Weight of the heaviest puppy in a litter	0.000	0.000
std of the puppy weights in a litter	0.333	
Litter size	0.128	
Parity	0.881	
Season of whelping	0.667	
Number of malpositioned fetuses, linear	0.000	0.000
Number of malpositioned fetuses, quadratic	0.000	0.042
Quality of the contractions	0.000	0.000
Inbreeding coefficient of the dam	0.973	
df	12	8
Akaike’s information criterion	896.69	858.23
Bayesian information criterion	950.73	894.25

The full model was calculated with an ordered logit regression. The final model was calculated with a generalized ordered logit regression. *P*-values are given for each independent variable in the model

**Fig. 1:**
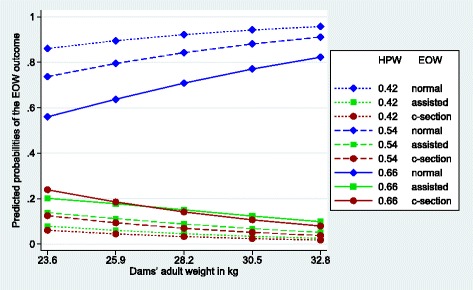
Predicted probabilities of EOW outcomes if contractions are normal and no malpositioned fetus is present. On the y-axis are the predicted probabilities of the nine outcomes of the target trait EOW for the weight of the dam ranging from the mean – 2sd (23.6 kg) to the mean + 2sd (32.8 kg) on the x-axis. HPW is the weight of the heaviest puppy in a litter in kg. All predicted probabilities are significantly different from zero

**Fig. 2:**
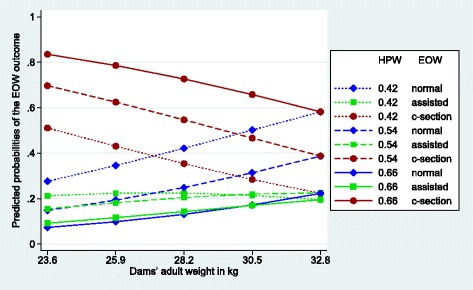
Predicted probabilities of EOW outcomes if contractions are poor and no malpositioned fetus is present. On the y-axis are the predicted probabilities of the nine outcomes of the target trait EOW for the weight of the dam ranging from the mean – 2sd (23.6 kg) to the mean + 2sd (32.8 kg) on the x-axis. HPW is the weight of the heaviest puppy in a litter in kg. All predicted probabilities are significantly different from zero with the exception of the estimate for a c-section at 32.8 kg in the case of a light heaviest puppy and the estimates at 23.6 kg in the case of a heavy heaviest puppy

**Fig. 3:**
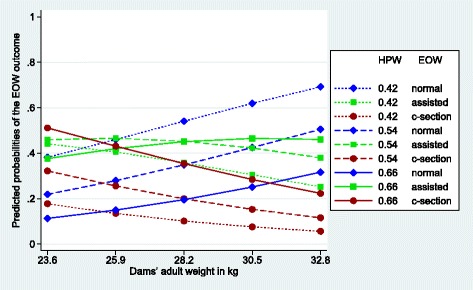
Predicted probabilities of EOW outcomes if contractions are normal and one fetus is malpositioned. On the y-axis are the predicted probabilities of the nine outcomes of the target trait EOW for the weight of the dam ranging from the mean – 2sd (23.6 kg) to the mean + 2sd (32.8 kg) on the x-axis. HPW is the weight of the heaviest puppy in a litter in kg. All predicted probabilities are significantly different from zero

**Fig. 4:**
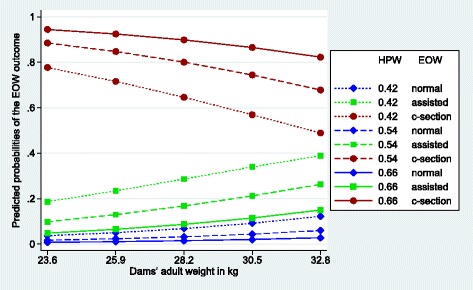
Predicted probabilities of EOW outcomes if contractions are poor and one fetus is malpositioned. On the y-axis are the predicted probabilities of the nine outcomes of the target trait EOW for the weight of the dam ranging from the mean – 2sd (23.6 kg) to the mean + 2sd (32.8 kg) on the x-axis. HPW is the weight of the heaviest puppy in a litter in kg. All predicted probabilities for a normal delivery, as well as for an assisted delivery of a heavy heaviest puppy, are not significantly different from zero whereas all others are

**Fig. 5:**
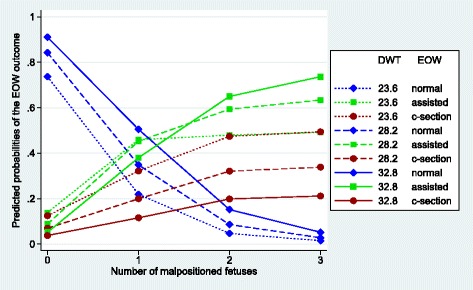
Predicted probabilities of EOW outcomes if contractions are normal and the weight of the heaviest puppy in a litter is 0.54 kg. On the y-axis are the predicted probabilities of the nine outcomes of the target trait EOW for the number of malpositioned fetuses ranging from 0 to 3 or more on the x-axis. DWT is the adult weight of the dam in kg. All predicted probabilities are significantly different from zero with the exception of a normal delivery in the case of 3 or more malpositioned fetuses, regardless of the weight of the dam

**Fig. 6:**
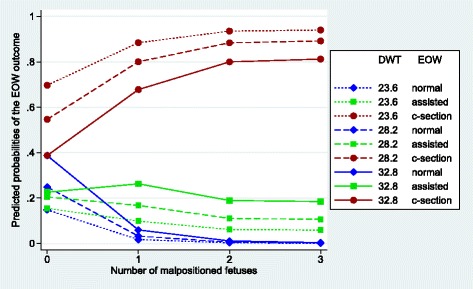
Predicted probabilities of EOW outcomes if contractions are poor and the weight of the heaviest puppy in a litter is 0.54 kg. On the y-axis are the predicted probabilities of the nine outcomes of the target trait EOW for the number of malpositioned fetuses ranging from 0 to 3 or more on the x-axis. DWT is the adult weight of the dam in kg. If the number of malpositioned fetuses is one or more, predicted probabilities for a normal delivery are not significantly different from zero, regardless of the weight of the dam. If the number of malpositioned fetuses is two or more, predicted probabilities for an assisted delivery are not significantly different from zero, regardless of the weight of the dam. All other predicted probabilities are significantly different from zero

Figures 1, 2, 3, 4, 5 and 6 visualize the probabilities of the three different outcomes of EOW for different representative combinations of the four predictors adult weight of the dam, weight of the heaviest puppy in a litter, number of malpositioned fetuses and quality of contractions. All predictions and their probabilities depicted in Figs. 1, 2, 3, 4, 5 and 6 are listed in Additional file 3.

Figure 1 shows the implications of an increasing adult weight of the dam for 3 different weights of the heaviest puppy in a litter in the case where the dams have normal contractions and no fetus is malpositioned. Actually, this constellation was observed in 507 litters, which is 76% of the total number of litters. Predicted probabilities for an assisted delivery and a c-section are very similar and low if the weight of the heaviest puppy in a litter is average or below average. If the weight of the heaviest puppy in a litter is high, these probabilities increase for both, but more steeply for a c-section, if the adult weight of the dam is low. The predicted probabilities of c-sections and assisted deliveries decrease slightly with an increasing adult weight of the dam. The differences between the extreme weights range from 0.04 probability points in the case of a c-section with a lightest weight of the heaviest puppy in a litter to 0.16 probability points in the case of a c-section with a heaviest weight of the heaviest puppy in a litter.

Figure 2 shows the huge impact of poor contractions on the probabilities of the c-section outcome in comparison to the situation in Fig. 1. The delivery conditions, poor contraction and no malpositioned fetus in a litter, are not very frequent in our data set (*n* = 14 or 2%). The risk for a c-section is highest (0.84) when the dam is light (23.6 kg) and the heaviest puppy of a litter is heavy (0.66 kg) that is 0.60 probability points higher than in Fig. 1. The decline of the risk of a c-section from light (23.6 kg) to heavy dams (32.8 kg) seems very similar for all 3 levels of the heaviest puppy in a litter (0.29, 0.31, and 0.25 probability points). Differences of probabilities of a c-section between light and heavy weights of the heaviest puppy in a litter are similar and vary over the whole range of the adult weight of the dam from 0.32 to 0.37 probability points. In contrast, the probabilities for an assisted whelping are only slightly higher as in Fig. 1 (0.12 probability points at an adult weight of the dam of 28.2 kg and a weight of the heaviest puppy in a litter of 0.54 kg). As a consequence of the high probabilities of a c-section, the probabilities of a normal whelping are much lower than in Fig. 1. The differences in the probabilities between light and heavy dams are more pronounced in Fig. 2.

Figure 3 shows that the impact of one malpositioned fetus is not as severe as poor contractions considering the predicted probability of a c-section. In our data, the combination of normal contractions and one malpositioned fetus occurred in 93 litters or 14% of the deliveries. Compared to Figs. 1 and 2 the probabilities for an assisted whelping are much higher (0.36 and 0.25 probability points, respectively) when the adult weight of the dam and the weight of the heaviest puppy in a litter are average. The risks for a c-section are higher than the ones in Fig. 1 but clearly lower than in Fig. 2 (0.13 and − 0.35 probability points, respectively) when the adult weight of the dam and the weight of the heaviest puppy in a litter are average. The probabilities of c-sections decrease with an increasing adult weight of the dam about twice as much for a heavy than for a light weight of the heaviest puppy in a litter (0.29 and 0.12 probability points, respectively).

Figure 4 shows an extremely unfavorable scenario where the dam has poor contractions and one fetus is malpositioned. Fortunately there were only 3 cases in our data set which makes about 0.4%. Comparing the curve patterns with those of Fig. 2 and Fig. 3, Fig. 4 is much more similar to Fig. 2 than to Fig. 3. This seems to confirm the impression from the comparison of Fig. 3 with Fig. 2, that poor contractions do affect whelping more than malpositioned fetuses. The probabilities for a normal delivery are all not significantly different from zero that is the weight of the heaviest puppy in a litter has no influence. If the weight of the heaviest puppy in a litter is heavy and the adult weight of the dam below average a c-section is practically unavoidable.

Figures 5 and 6 show the influence of the number of malpositioned fetuses for 3 different adult weights of the dam on the outcome of EOW in the case where the weight of the heaviest puppy in a litter is average (0.54 kg).

Figure 5 shows the situation for dams with normal contractions which was observed in 97% of our data. Probabilities for a normal delivery drop to or close to zero if there is more than one malpositioned fetus. In the case of 3 or more malpositioned fetuses the predicted probabilities for a normal delivery for all three levels of the adult weight of the dam are not significantly different from zero. In the case of one malpositioned fetus, the probability for a normal delivery is much higher in the case of a heavy adult weight of the dam (0.51) in comparison to a light adult weight of the dam (0.22). The increase of the risk for a c-section from 0 to 2 malpositioned fetuses is linear but much greater for a light than for a heavy adult weight of the dam (0.35 and 0.16 probability points, respectively). The increase of that risk between 2 and 3 malpositioned fetuses is much smaller (0.02 and 0.01 probability points, respectively). The increase of the risk of assisted deliveries from 0 to 2 malpositioned fetuses is similar for a heavy and average adult weight of the dam (0.60 and 0.51 probability points, respectively), whereas the increase from 2 to 3 is clearly smaller (0.09 and 0.04 probability points, respectively). For a light adult weight of the dam the risk of assisted delivery increased by 0.32 probability points when the number of malpositioned fetuses increased from zero to one, with no real increase thereafter (0.03 probability points), when the number of malpositioned fetuses increased from one to three or more.

Figure 6 shows the situation where the dam has poor contractions. This situation was observed only 22 times in our data set or in 3% of all cases. It is apparent that the 3 c-section curves are positioned at much higher predicted probabilities in comparison to Fig. 5, e.g. the increase at 0 malpositioned fetuses for an average adult weight of the dam (28.2 kg) amount to 0.48 probability points and at 1 malpositioned fetus to 0.60 probability points. A c-section practically is unavoidable in the case of a light dam and 2 or more malpositioned fetuses. The probabilities of assisted deliveries decrease marginally from 0 to 3 or more malpositioned fetuses but the range between a light and heavy adult weight of the dam are smallest at 0 with 0.07 probability points and about double at the other malposition levels with 0.13 to 0.17 probability points.

## Discussion

Dystocia is a common emergency in dogs and criteria for the decision to perform a timely c-section have been proposed [10]. Most of the dystocia cases in dogs are of maternal origin [1]. In a multicenter study in the USA and Canada the LR was among the top five breeds for emergency and elective c-sections during 1994 to 1997 [12]. In a multi-breed study c-sections were performed in 20% of the parturitions in LR [4]. c-sections bring a health risk for both the dam and the puppies. Therefore, the reduction of emergency c-sections is desirable in dog breeding to enhance animal welfare.

The comprehensive and large data set for parturitions recorded by the Guiding Eyes for the Blind (Additional file 4) allows for the analysis of possible predictors for c-sections. In the present data set there was one heaviest puppy weight, 0.11 kg (weight of the dam 25.40 kg) that could be considered an extreme value. Plotting the leverage against the normalized residual squared clearly showed that it did have a large residual but no leverage meaning that it had no great impact on the estimates of the regression coefficients (Additional file 1). The weight of the dams showed no extreme values. Neither the dam nor the sire could significantly explain part of the total variance in our data. It is not surprising that the body mass index of the dams did not affect the probability of the occurrence of a c-section as the body mass index in dogs probably does not reflect the condition of the body as it does it in humans. The Guiding Eyes breeding stock are also closely monitored and managed to maintain their established target weight within ±1.5 kg. We hypothesized that the weight to height ratio characterizes the body condition of a dog much better than the body mass index. Also we considered the weight to height ratio a better metric than the weight as the weighting with the height at withers will bring extreme values of the weight nearer to the mean. The fact that the weight to height ratio did not stay in the model probably is due to missing measurements of the height that led to a loss of about one third of the observations in comparison to weight. The inbreeding coefficient of the dam had no impact on the probability of the occurrence of a c-section, which may be due to the relatively low inbreeding rates with two thirds of the dams having an inbreeding coefficient below 10%. There was no influence of the whelping season. The seasonal occurrence of c-sections ranged from 11% (fall) over 14% (spring) and 15% (summer) to 16% (winter). Also the parity had no influence. Looking at the 6 parities, the percentage of c-sections is more or less evenly distributed over parities, ranging from 14% in parity 3 to 18% in parity 4 with the exception of parities 2 and 6 with only 9% c-sections. Of the litter characteristics, the weight of the heaviest puppy had an impact on the probability of the occurrence of a c-section. It is plausible that a heavier puppy is a greater physical challenge to the whelping dam than a lighter puppy, due to feto-maternal disproportion. The influence of the observer, here the whelpers, on the observations as described in other studies [13, 14] was not taken into account because whelpers were assigned to whelpings according to a schedule and had to follow a clear protocol. Therefore, the occurrence of c-sections does not reflect the presence of specific whelpers but rather is the consequence of the procedure in the protocol. The duration of a whelping was not considered in the analyses because it rather seems to be a consequence of whelping problems than its source. Also the number of stillborn puppies was not considered as a predictor because the death of a puppy could also be caused by factors leading to a c-section, e.g. suffocation during a prolonged whelping.

Our data confirms that poor contractions of a dam, as an indicator for uterine inertia, as well as malpositioned fetuses increase the probability that a whelping has to be assisted or that a c-section cannot be avoided. These factors can be related because contracting against a malpositioned fetus can lead to uterine inertia from exhaustion of uterine muscles. Malpositioned fetuses are likely the result of a feto-maternal disproportion or maternal characteristics resulting in the vaginal vault being too small for the fetus to reposition itself. Our study allowed for the examination of two additional factors leading to c-sections, the weight of the dam and the weight of the heaviest puppy in a litter. Although not significant, dams at first parity are with an average weight of 28.07 kg lighter than dams at later parities with an average weight of 28.31 kg. Dams at second or higher parity with a normal delivery on average were 0.60 kg heavier than dams with a c-section that weigh on average 27.91 kg. In the case of the first parity they are on average 1.46 kg heavier. The weight of the dams certainly has a genetic background. In another colony of LR at The Seeing Eye in New Jersey, heritabilities were estimated to be 0.44 for adult weight and 0.46 for adult height [15]. The influence of the birthweight on the probability of requiring assistance at delivery or a c-section was expected as this phenomenon is known in cattle (e.g. [16, 17]). Besides direct genetic effects, paternal and especially maternal genetic effects could, as in cattle, also play a role in dog. The genetic correlation between pelvic opening and the maternal genetic effect of dystocia scores in cattle is high with − 0.62 in heifers and − 0.77 in cows [18]. Heritabilities of direct and maternal effects were 0.25 and 0.08 respectively while heritability of cow pelvic opening was 0.21. Other authors obtained even larger heritabilities for pelvic opening with h^2^ = 0.42 [19].

## Conclusions

The four predictors, poor contractions, number of malpositioned fetuses, weight of the dam and the weight of the heaviest puppy in a litter, had an impact on the probability of the occurrence of a c-section in LR. Therefore, the consideration of the adult weight of the dam may reduce the risk of c-sections in this colony. Dams with an adult body weight substantially below average should not be selected as breeders in this colony. The problem of the weight of the heaviest puppy in a litter must be tackled on the genetic track resorting to the estimation of breeding values that allow for maternal and paternal effects.

Our findings apply to this colony of LR and are not necessarily, but could be, also meaningful for breeds of similar stature at large. To evaluate the impact of our results on the general LR population data on the weights of the puppies at birth and the weight and possibly the height of the dam at mating are needed. Whether our findings help to improve the situation with respect to c-sections in guide dogs depends heavily on how they can be implemented in a breeding strategy that is focused on the guiding abilities of the dogs.

## Additional files

Additional file 1: Presenting the model development for the analysis of EOW. (DOCX 531 kb)

Additional file 2: Presenting descriptive statistics for all variables in the analyses. (DOCX 27 kb)

Additional file 3: Displaying for Figs. 1, 2, 3, 4, 5 and 6 the predicted probabilities for the EOW outcomes, together with the *P*-values for the tests of predicted probabilities being different from zero. (DOCX 31 kb)

Additional file 4: Displaying the data analyzed. (XLSX 111 kb)

## Abbreviations

EOW: Ease of whelping
IR: Initial Rise
LR: Labrador Retriever
