# Supplementary material for: Factors contributing to the decision to perform a cesarean section in Labrador retrievers
Source: BMC Vet Res. 2018 Feb 27;14:57. doi: 10.1186/s12917-018-1381-8 (PMC5828337; doi:10.1186/s12917-018-1381-8)
Supplement: Supplementary file 1 — Presenting the model development for the analysis of EOW. (DOCX 531 kb) [file 12917_2018_1381_MOESM1_ESM.docx]

**Additional file 1: Statistical Analyses of the ease of whelping**

Statistical analyses were carried out using Stata/SE 14.1 (StataCorp, 4905 Lakeway Drive, College Station, Texas 77845, USA). The initial data set comprised 667 litters of LR from 2003 to 2016 by 255 different dams and 148 sires. If not stated differently the significance level for statistical tests is 0.05.

The target trait (**EOW**), ease of whelping, was ordinal with the three levels “normal whelping”, “assisted whelping without C-section” and “C-section with or without prior assistance”.

The following variables were evaluated as fixed effects and possible predictors for the outcomes of EOW: Parity, number of malpositioned fetuses, quality of the contractions, whelping season, inbreeding coefficient of the dam, adult weight of the dam, height at withers of the dam, body mass index of the dam, weight to height ratio of the dam, size of the litter, weight of the litter, average puppy weight, standard deviation of the puppy weight in a litter, variance of the puppy weights in a litter, and weight of the heaviest puppy in a litter. All variables are described in Supplement 2.

In addition the identities of the dams and sires were analysed as random effects.

**Correlations and selection of predictors**

Correlation coefficients between possible predictors were calculated to avoid the inclusion of highly correlated predictors. Specifically two groups of variables were looked at, one group describing the body condition of the dam and the other the litter.

**Variables describing the body condition of the dam**, 464 observations:

|  | 1 | 2 | 3 | 4 |
| --- | --- | --- | --- | --- |
| 1 Body mass index kg/m2 | 1.00 |  |  |  |
| 2 Height at withers cm | -0.61 | 1.00 |  |  |
| 3 Adult weight kg | 0.32 | 0.55 | 1.00 |  |
| 4 Weight to height ratio kg/cm | 0.82 | -0.05 | 0.81 | 1.00 |

With the exception of the height at withers and the weight to height ratio all the metrics are moderately to highly correlated among themselves.

Variables describing the body condition of the dam were evaluated as predictors for EOW using ordered logistic regression (Stata command: ologit). The two cuts or thresholds of EOW were estimated in all models. For all models throughout all analyses, estimates were stored (Stata command: estimates store) and comparisons of the models (Stata command: lrtest model_x model_y, stats) were based on both, Akaike’s information criterion (AIC) [1] and the Bayesian information criterion (BIC) [2].

| Model | Structure | n | df | AIC | BIC |
| --- | --- | --- | --- | --- | --- |
| 0 | Intercept only | 464 | 2 | 788.9 | 797.1 |
| 1 | Body mass index | 464 | 3 | 789.6 | 802.0 |
| 2 | Height at withers | 464 | 3 | 788.2 | 800.6 |
| 3 | Adult weight | 464 | 3 | 780.2 | 792.6 |
| 4 | Weight to height ratio | 464 | 3 | 784.0 | 796.5 |

Clearly, the weight of the dam fits the data best. In the following analyses, only the weight of the dam was considered as all the other body condition metrics were correlated with the weight of the dam.

**Variables describing the litter**, 667 observations:

|  | 1 | 2 | 3 | 4 | 5 | 6 |
| --- | --- | --- | --- | --- | --- | --- |
| 1 Average puppy weight | 1.00 |  |  |  |  |  |
| 2 Heaviest puppy weight | 0.85 | 1.00 |  |  |  |  |
| 3 Size of a litter | -0.45 | -0.33 | 1.00 |  |  |  |
| 4 weight of a litter | -0.05 | 0.03 | 0.90 | 1.00 |  |  |
| 5 Std of the puppy weight of a litter | -0.12 | 0.22 | -0.05 | -0.11 | 1.00 |  |
| 6 Variance of the puppy weight of a litter | -0.14 | 0.15 | -0.06 | -0.14 | 0.94 | 1.00 |

There are three pairs of metrics that are highly correlated:

1. the average puppy weight and the weight of the heaviest puppy in a litter
2. the size and weight of a litter
3. the standard deviation and the variance of the litter.

We chose to use the better predictor of the three groups in the analyses that follow, so that there were three predictors characterizing the litter in the full model.

Variables describing the litter were evaluated as predictors for EOW using ordered logistic regression (Stata command: ologit). The two cuts or thresholds of EOW were estimated in all models.

| Model | Structure | n | df | AIC | BIC |
| --- | --- | --- | --- | --- | --- |
| 00 | Intercept only | 667 | 2 | 1117.9 | 1126.9 |
| 01 | Average puppy weight | 667 | 3 | 1090.2 | 1103.7 |
| 02 | Weight heaviest puppy | 667 | 3 | 1079.0 | 1092.5 |
| 03 | Litter size | 667 | 3 | 1116.4 | 1129.9 |
| 04 | Litter weight | 667 | 3 | 1119.7 | 1133.2 |
| 05 | Std puppy weight | 667 | 3 | 1115.1 | 1128.6 |
| 06 | Variance puppy weight | 667 | 3 | 1116.5 | 1130.0 |

The better predictors for the outcomes of EOW of the three groups were the weight of the heaviest puppy, the litter size and the standard deviation of the puppy weight in a litter.

**Evaluation of random effects**

To evaluate whether the dam and/or the sire influence the outcomes of EOW, models with just the dam and/or sire were analyzed using multilevel ordered logistic regression (Stata command: meologit) and compared with the empty model. This approach was chosen because multilevel ordered logistic regression with several predictors and more than one random effect tend to run very slow or often, feasible initial values cannot be found. The two cuts or thresholds of EOW were estimated in all models.

| Model | Structure | n | df | AIC | BIC |
| --- | --- | --- | --- | --- | --- |
| 000 | Intercept only | 667 | 2 | 1117.856 | 1126.862 |
| 001 | Dam & sire | 667 | 4 | 1103.159 | 1121.170 |
| 002 | Dam only | 667 | 3 | 1099.365 | 1112.873 |
| 003 | Sire only | 667 | 3 | 1114.424 | 1127.933 |

The model with only the dam as random effect clearly fits the data best. As we now only had one random effect in the model, we ran a model including all selected fixed and random effects:

| Fixed effects | Adult weight of the dam kg |
| --- | --- |
|  | Weight of the heaviest puppy in a litter kg |
|  | Std of the weights of the puppies in a litter kg |
|  | Litter size, ranging from 2 to 13 |
|  | Parity, ranging from 1 to 6 |
|  | Season with levels ranging from 1 to 4 |
|  | Number of malpositioned fetuses, ranging from 0 to 3 |
|  | (Number of malpositioned fetuses) squared |
|  | Quality of the contractions with levels 0 and 1 |
|  | Inbreeding coefficient of the dam |
| Random effects | Dam |

The quadratic term for the number of malpositioned fetuses was introduced because we suspected based on the distribution of the observations over the 4 classes that only a linear term may not sufficiently explain the data.

| Variables | Coefficients | Standard Error | P-value |
| --- | --- | --- | --- |
| Adult weight dam | -0.163 | 0.053 | 0.002 |
| Weight heaviest puppy | 7.769 | 2.037 | 0.000 |
| Std of puppy weights | 3.779 | 3.917 | 0.335 |
| Litter size | 0.083 | 0.055 | 0.131 |
| Parity | 0.011 | 0.079 | 0.884 |
| Season | 0.037 | 0.086 | 0.665 |
| Num. malpositioned fetuses | 2.469 | 0.317 | 0.000 |
| (Num. malpositioned fetuses)^2^ | -0.517 | 0.118 | 0.000 |
| Quality contractions | 3.082 | 0.582 | 0.000 |
| Inbreeding coefficient dam | 0.080 | 2.777 | 0.977 |
| Dam (variance component) | 0.034 | 0.236 | - |

With a standard error almost 7 times as large as the estimated mean, the variance component of the dam is not different from zero and was dropped from the following models.

**Model evaluation for EOW**

All models were calculated with 667 observations using ordered logistic regression (Stata command: ologit).

**Model 0001** (full model)

| Variables | Coefficients | Standard Error | P-value |
| --- | --- | --- | --- |
| Adult weight dam | -0.161 | 0.050 | 0.001 |
| Weight heaviest puppy | 7.710 | 1.976 | 0.000 |
| Std of puppy weights | 3.768 | 3.889 | 0.333 |
| Litter size | 0.082 | 0.054 | 0.128 |
| Parity | 0.012 | 0.078 | 0.881 |
| Season | 0.037 | 0.086 | 0.667 |
| Num. malpositioned fetuses | 2.465 | 0.314 | 0.000 |
| (Num. malpositioned fetuses)^2^ | -0.518 | 0.117 | 0.000 |
| Quality contractions | 3.075 | 0.576 | 0.000 |
| Inbreeding coefficient dam | 0.092 | 2.745 | 0.973 |

For the next model the inbreeding coefficient of the dam was dropped.

**Model 0002**

| Variables | Coefficients | Standard Error | P-value |
| --- | --- | --- | --- |
| Adult weight dam | -0.161 | 0.050 | 0.001 |
| Weight heaviest puppy | 7.719 | 1.957 | 0.000 |
| Std of puppy weights | 3.757 | 3.876 | 0.332 |
| Litter size | 0.082 | 0.054 | 0.128 |
| Parity | 0.011 | 0.086 | 0.883 |
| Season | 0.037 | 0.086 | 0.666 |
| Num. malpositioned fetuses | 2.466 | 0.314 | 0.000 |
| (Num. malpositioned fetuses)^2^ | -0.518 | 0.117 | 0.000 |
| Quality contractions | 3.078 | 0.572 | 0.000 |

| Model | Structure | n | df | AIC | BIC |
| --- | --- | --- | --- | --- | --- |
| 0001 | Full | 667 | 12 | 896.7 | 950.7 |
| 0002 | Inbreeding coefficient dam dropped | 667 | 11 | 894.7 | 944.2 |

Model 0002 fits the data better than model 0001. For the next model the parity was dropped.

**Model 0003**

| Variables | Coefficients | Standard Error | P-value |
| --- | --- | --- | --- |
| Adult weight dam | -0.160 | 0.049 | 0.001 |
| Weight heaviest puppy | 7.706 | 1.955 | 0.000 |
| Std of puppy weights | 3.762 | 3.875 | 0.332 |
| Litter size | 0.081 | 0.054 | 0.130 |
| Season | 0.036 | 0.086 | 0.670 |
| Num. malpositioned fetuses | 2.471 | 0.312 | 0.000 |
| (Num. malpositioned fetuses)^2^ | -0.520 | 0.116 | 0.000 |
| Quality contractions | 3.076 | 0.572 | 0.000 |

| Model | Structure | n | df | AIC | BIC |
| --- | --- | --- | --- | --- | --- |
| 0001 | Full | 667 | 12 | 896.7 | 950.7 |
| 0002 | Inbreeding coefficient dam dropped | 667 | 11 | 894.7 | 944.2 |
| 0003 | Parity dropped | 667 | 10 | 892.7 | 937.7 |

Model 0003 fits the data better than model 0002. For the next model the season was dropped.

**Model 0004**

| Variables | Coefficients | Standard Error | P-value |
| --- | --- | --- | --- |
| Adult weight dam | -0.160 | 0.049 | 0.001 |
| Weight heaviest puppy | 7.632 | 1.945 | 0.000 |
| Std of puppy weights | 3.597 | 3.852 | 0.350 |
| Litter size | 0.080 | 0.054 | 0.134 |
| Num. malpositioned fetuses | 2.464 | 0.311 | 0.000 |
| (Num. malpositioned fetuses)^2^ | -0.518 | 0.116 | 0.000 |
| Quality contractions | 3.066 | 0.571 | 0.000 |

| Model | Structure | n | df | AIC | BIC |
| --- | --- | --- | --- | --- | --- |
| 0001 | Full | 667 | 12 | 896.7 | 950.7 |
| 0002 | Inbreeding coefficient dam dropped | 667 | 11 | 894.7 | 944.2 |
| 0003 | Parity dropped | 667 | 10 | 892.7 | 937.7 |
| 0004 | Season dropped | 667 | 9 | 890.9 | 931.4 |

Model 0004 fits the data better than model 0003. For the next model the standard deviation of the puppy weights in a litter was dropped.

**Model 0005**

| Variables | Coefficients | Standard Error | P-value |
| --- | --- | --- | --- |
| Adult weight dam | -0.162 | 0.049 | 0.001 |
| Weight heaviest puppy | 8.007 | 1.909 | 0.000 |
| Litter size | 0.083 | 0.053 | 0.120 |
| Num. malpositioned fetuses | 2.465 | 0.311 | 0.000 |
| (Num. malpositioned fetuses)^2^ | -0.521 | 0.116 | 0.000 |
| Quality contractions | 3.097 | 0.571 | 0.000 |

| Model | Structure | n | df | AIC | BIC |
| --- | --- | --- | --- | --- | --- |
| 0001 | Full | 667 | 12 | 896.7 | 950.7 |
| 0002 | Inbreeding coefficient dam dropped | 667 | 11 | 894.7 | 944.2 |
| 0003 | Parity dropped | 667 | 10 | 892.7 | 937.7 |
| 0004 | Season dropped | 667 | 9 | 890.9 | 931.4 |
| 0005 | Std of puppy weights dropped | 667 | 8 | 889.8 | 925.8 |

Model 0005 fits the data better than model 0004. For the next model the size of a litter was dropped.

**Model 0006**

| Variables | Coefficients | Standard Error | P-value |
| --- | --- | --- | --- |
| Adult weight dam | -0.135 | 0.046 | 0.003 |
| Weight heaviest puppy | 6.776 | 1.723 | 0.000 |
| Num. malpositioned fetuses | 2.482 | 0.311 | 0.000 |
| (Num. malpositioned fetuses)^2^ | -0.522 | 0.116 | 0.000 |
| Quality contractions | 3.060 | 0.570 | 0.000 |

| Model | Structure | n | df | AIC | BIC |
| --- | --- | --- | --- | --- | --- |
| 0001 | Full | 667 | 12 | 896.7 | 950.7 |
| 0002 | Inbreeding coefficient dam dropped | 667 | 11 | 894.7 | 944.2 |
| 0003 | Parity dropped | 667 | 10 | 892.7 | 937.7 |
| 0004 | Season dropped | 667 | 9 | 890.9 | 931.4 |
| 0005 | Std of puppy weights dropped | 667 | 8 | 889.8 | 925.8 |
| 0006 | Litter size dropped | 667 | 7 | 890.2 | 921.7 |

Although AIC is ambivalent, BIC favors model 0006. All estimates of coefficients are significantly different from zero. Therefore model 0006 was our final model.

**Checking for outliers**

At this point the two quantitative predictors, the weight of the dam and the weight of the heaviest puppy in a litter, were checked for outliers which could have influenced the outcome of the different models.

The 5 largest and the 5 smallest values with the corresponding frequencies were identified (Stata command: fre) [3].

| Weight dam kg | Frequency |
| --- | --- |
| 22.68 | 2 |
| 23.13 | 1 |
| 23.59 | 6 |
| 24.04 | 14 |
| 24.49 | 10 |
|  |  |
| 33.57 | 1 |
| 34.01 | 3 |
| 34.47 | 7 |
| 36.29 | 1 |
| 36.74 | 3 |

The adult weight of the dam appears to come in bins, which probably is because we have only 256 dams that were weighed in pounds without decimals. Pounds then were transformed into kilograms and rounded. None of the weights of the dam seems to be an outlier.

| Weight heaviest puppy kg | Frequency |
| --- | --- |
| 0.11 | 1 |
| 0.39 | 1 |
| 0.40 | 1 |
| 0.41 | 3 |
| 0.42 | 2 |
|  |  |
| 0.67 | 2 |
| 0.68 | 6 |
| 0.69 | 2 |
| 0.71 | 3 |
| 0.77 | 1 |

The lightest of the heaviest puppy in a litter, with only 0.11 kg, seems to be an outlier.

A leverage versus squared residuals plot visualizes the potential impact of outliers on the estimates of a linear regression. A regression analysis of the weight of the heaviest puppy in a litter against the weight of the dam was performed (Stata command: regress), followed by such a leverage plot (Stata command: lvr2plot) [4].


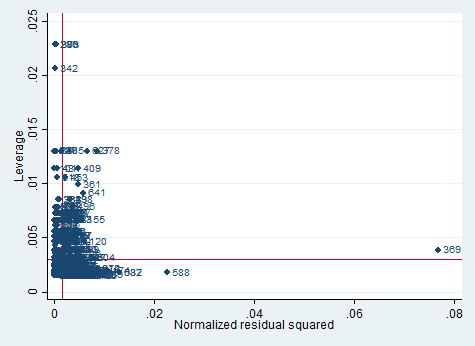


The horizontal red line shows the average of the normalized residuals squared and the vertical line the average of leverage. Observations 369 has a large residual but practically no leverage. Observations in the upper right of the graph would be worrisome because they would have both, high leverage and large residuals. Leverage is a measure of how far away the independent variable values of an observation are from those of the other observations. To explain the meaning of leverage often the formula “influence on coefficients = leverage * discrepancy” is given. In our case, this observation has no great impact on the estimate of the regression coefficient. Besides that, we know of no reason why this observation should have been excluded, so it stayed in the analyses and we kept model0006 as our final model.

**Testing for violations of the parallel regression lines assumption**

We checked for model 0006 whether the parallel regression lines assumption was violated for the individual variables (Stata command: brant) [5].

| Variable | Chisq | P-value | df |
| --- | --- | --- | --- |
| Weight dam | 0.66 | 0.418 | 1 |
| Weight heaviest puppy | 3.41 | 0.065 | 1 |
| Num. malpositioned fetuses | 1.61 | 0.205 | 1 |
| (Num. malpositioned fetuses)^2^ | 0.49 | 0.483 | 1 |
| Quality contractions | 1.38 | 0.241 | 1 |

The P-values indicated that the coefficients for going from EOW level normal to the combined levels assisted and c-section are not different from the corresponding ones for going from the combined levels normal and assisted to level c-section.

In the next step we compared the ordered logit model with the fully generalized ordered logit model, which relaxes the parallel regression assumption on all explanatory variables (Stata command: oparallel) [6]. In this step we also obtained AIC and BIC for the two models.

| Test | Chisq | df | P-value |
| --- | --- | --- | --- |
| Wolfe Gould | 37.55 | 5 | 0.000 |
| Brant | 25.76 | 5 | 0.000 |
| Score | 34.15 | 5 | 0.000 |
| Likelihood ratio | 36.66 | 5 | 0.000 |
| Wald | 24.59 | 5 | 0.000 |

| Criterion | ologit | gologit | Difference |
| --- | --- | --- | --- |
| AIC | 890.20 | 863.55 | 26.22 |
| BIC | 921.72 | 917.58 | 4.14 |

All five test statistics say that the parallel lines assumption is violated. AIC and BIC clearly show that gologit (number of levels -1 coefficients for each independent variable) makes a better fit than ologit (1 coefficient for each independent variable). Therefore model 0006, our final model, was re-analyzed with a generalized ordered logit regression (Stata command: gologit2) with the option of relaxing the parallel regression assumption for violating variables [7]. As no constraints were imposed for the number of malpositioned fetuses the output is presented in two parts.

1

| Variables | Coefficients | Standard Error | P-value |
| --- | --- | --- | --- |
| Adult weight dam | -0.140 | 0.047 | 0.003 |
| Weight heaviest puppy | 6.576 | 1.749 | 0.000 |
| Num. malpositioned fetuses | 2.582 | 0.325 | 0.000 |
| (Num. malpositioned fetuses)^2^ | -0.281 | 0.138 | 0.042 |
| Quality contractions | 2.780 | 0.547 | 0.000 |

2

| Variables | Coefficients | Standard Error | P-value |
| --- | --- | --- | --- |
| Adult weight dam | -0.140 | 0.047 | 0.003 |
| Weight heaviest puppy | 6.576 | 1.749 | 0.000 |
| Num. malpositioned fetuses | 1.483 | 0.377 | 0.000 |
| (Num. malpositioned fetuses)^2^ | -0.281 | 0.138 | 0.042 |
| Quality contractions | 2.780 | 0.547 | 0.000 |

The linear term for the number of malpositioned fetuses has two different estimates for the coefficient, the first from comparing EOW level normal against assisted and c-section combined, and the second from comparing normal and assisted combined against c-section.

AIC and BIC were estimated for this model (Stata command: estat ic)

| Model | Structure | n | df | AIC | BIC |
| --- | --- | --- | --- | --- | --- |
| gologit2 | Final | 667 | 8 | 858.2 | 894.2 |

AIC and BIC are better than the ones for gologit in the previous comparison between ologit and gologit because here constraints were imposed for the weight of the dam, the weight of the heaviest puppy of a litter and the quality of the contractions.

**Generation of Figures 1 to 6**

The coefficients estimated refer to the logit scale and not to the observational scale. To interpret our results we calculated and plotted predicted probabilities for meaningful data points as recommended by Long and Freese [8] (Stata command: margins, plot). Meaningful data points in our analyses were the mean (28.2 kg) and 1 (± 2.3 kg) or 2 (± 4.6 kg) standard deviations for the weight of the dam, the mean (0.54 kg) and 2 standard deviations (± 0.12 kg) for the weight of the heaviest puppy in a litter, all 4 levels for the number of malpositioned fetuses in a litter, and both levels for the quality of the contractions of the dam.

**Literature**

1. Akaike H. A new look at the statistical model identification. IEEE Trans Autom Control. 1974; AC-19: 716-23.

2. Schwarz G. Estimating the dimension of a model. 1978; Ann Stat. 6; 461-4.

3. Jann B. fre: Stata module to display one-way frequency table. 2007; available from <http://ideas.repec.org/c/boc/bocode/s456835.html>.

4. Mallows CL. Augmented partial residuals. 1986; Technometrics 28, 313-9.

5. Brant R. Assessing proportionality in the proportional odds model for ordinal logistic regression. 1990; Biometrics. 46: 1171-8.

6. Buis ML. OPARALLEL: Stata module providing post-estimation command for testing the parallel regression assumption. 2013; available from <https://ideas.repec.org/c/boc/bocode/s457720.html>.

7. Williams R. Generalized ordered logit/partial proportional odds models for ordinal dependent variables. 2006; The Stata Journal. 6: 58-82.

8. 14. Long JS, Freese J. Regression models for categorical dependent variables using Stata. 3rd ed. College Station, TX, USA: Stata Press; 2014.
