# Supplementary material for: Factors contributing to the decision to perform a cesarean section in Labrador retrievers
Source: BMC Vet Res. 2018 Feb 27;14:57. doi: 10.1186/s12917-018-1381-8 (PMC5828337; doi:10.1186/s12917-018-1381-8)
Supplement: Supplementary file 2 — Presenting descriptive statistics for all variables in the analyses. (DOCX 27 kb) [file 12917_2018_1381_MOESM2_ESM.docx]

**Additional file 2: Descriptive statistics**

Number of litters with respect of the ease of whelping and the categorical traits evaluated in the analyses. Percentages are column percent.

|  | **Normal** | | **Assisted** | | **C-section** | | **Total** | |
| --- | --- | --- | --- | --- | --- | --- | --- | --- |
| **Number of litters** | 459 | | 115 | | 93 | | 667 | |
|  | **N** | **%** | **N** | **%** | **N** | **%** | **N** | **%** |
| **Parity** | | | | | | | | |
| 1 | 175 | 38.13 | 33 | 28.70 | 43 | 46.24 | 251 | 37.63 |
| 2 | 132 | 28.76 | 38 | 33.04 | 16 | 17.20 | 186 | 27.89 |
| 3 | 78 | 16.99 | 24 | 20.87 | 16 | 17.20 | 118 | 17.69 |
| 4 | 47 | 10.24 | 13 | 11.30 | 13 | 13.98 | 73 | 10.94 |
| 5 | 17 | 3.70 | 7 | 6.09 | 4 | 4.30 | 28 | 4.20 |
| 6 | 10 | 2.18 | 0 | 0.00 | 1 | 1.08 | 11 | 1.65 |
| **Number of malpositioned fetuses** | | | | | | | | |
| 0 | 426 | 92.81 | 47 | 40.87 | 47 | 50.54 | 520 | 77.96 |
| 1 | 31 | 6.75 | 44 | 38.26 | 21 | 22.58 | 96 | 14.39 |
| 2 | 2 | 0.44 | 10 | 8.70 | 16 | 17.20 | 28 | 4.20 |
| 3 or more | 0 | 0.00 | 14 | 12.17 | 9 | 9.68 | 23 | 3.45 |
| **Quality of the contractions** | | | | | | | | |
| normal (0) | 456 | 99.35 | 113 | 98.26 | 76 | 81.72 | 645 | 96.70 |
| poor (1) | 3 | 0.65 | 2 | 1.74 | 17 | 18.28 | 22 | 3.30 |
| **Season of whelping** | | | | | | | | |
| Jan to Mar (1) | 120 | 26.14 | 26 | 22.61 | 28 | 30.11 | 174 | 26.09 |
| Apr to Jun (2) | 109 | 23.75 | 29 | 25.22 | 23 | 24.73 | 161 | 24.14 |
| Jul to Sep (3) | 101 | 22.00 | 38 | 33.04 | 25 | 26.88 | 164 | 24.59 |
| Oct to Dec (4) | 129 | 28.10 | 22 | 19.13 | 17 | 18.28 | 168 | 25.19 |
| **Size of a litter** | | | | | | | | |
| 2 | 5 | 1.09 | 0 | 0.00 | 0 | 0.00 | 5 | 0.75 |
| 3 | 6 | 1.31 | 3 | 2.61 | 4 | 4.30 | 13 | 1.95 |
| 4 | 9 | 1.96 | 2 | 1.74 | 3 | 3.23 | 14 | 2.19 |
| 5 | 27 | 5.88 | 12 | 10.43 | 11 | 11.83 | 50 | 7.50 |
| 6 | 53 | 11.55 | 12 | 10.43 | 11 | 11.83 | 76 | 11.39 |
| 7 | 71 | 15.47 | 22 | 19.13 | 10 | 10.75 | 103 | 15.44 |
| 8 | 95 | 20.70 | 26 | 22.61 | 15 | 16.13 | 136 | 20.39 |
| 9 | 84 | 18.30 | 13 | 11.30 | 18 | 19.35 | 115 | 17.24 |
| 10 | 62 | 13.51 | 17 | 14.78 | 14 | 15.05 | 93 | 13.94 |
| 11 | 28 | 6.10 | 7 | 6.09 | 3 | 3.23 | 38 | 5.70 |
| 12 | 15 | 3.27 | 1 | 0.87 | 4 | 4.30 | 20 | 3.00 |
| 13 | 4 | 0.87 | 0 | 0.00 | 0 | 0.00 | 4 | 0.60 |

Number of litters, mean, standard deviation and range of the continuous dam variables evaluated in the analyses with respect to the ease of whelping.

|  | **Normal** | **Assisted** | **C-section** | **Total** |
| --- | --- | --- | --- | --- |
| **Inbreeding coefficient of the dam** | | | | |
| N | 459 | 115 | 93 | 667 |
| Mean | 0.076 | 0.090 | 0.083 | 0.079 |
| Std Dev | 0.034 | 0.035 | 0.033 | 0.035 |
| Range | 0.000 – 0.181 | 0.000 – 0.169 | 0.000 – 0.0167 | 0.000 – 0.181 |
| **Adult weight of the dam in kg** | | | | |
| N | 459 | 115 | 93 | 667 |
| Mean | 28.48 | 27.80 | 27.48 | 28.22 |
| Std Dev | 2.31 | 2.01 | 2.08 | 2.26 |
| Range | 22.68 – 36.74 | 22.68 – 32.66 | 23.13 – 34.47 | 22.68 – 36.74 |
| **Height at withers of the dam in cm** | | | | |
| N | 316 | 81 | 67 | 464 |
| Mean | 55.9 | 55.5 | 55.5 | 55.8 |
| Std Dev | 2.5 | 2.3 | 2.4 | 2.5 |
| Range | 48.3 – 63.5 | 48.3 – 60.5 | 50.5 – 60.5 | 48.3 – 63.5 |
| **Body mass index of the dam in kg/m^2^** | | | | |
| N | 316 | 81 | 67 | 464 |
| Mean | 90.9 | 90.5 | 89.7 | 90.6 |
| Std Dev | 6.9 | 8.2 | 7.4 | 7.2 |
| Range | 74.1 – 111.0 | 74.2 – 111.0 | 73.1 – 109.0 | 73.1 – 111.0 |
| **Weight to height ratio of the dam in kg/cm** | | | | |
| N | 316 | 81 | 67 | 464 |
| Mean | 0.507 | 0.501 | 0.497 | 0.504 |
| Std Dev | 0.031 | 0.035 | 0.032 | 0.032 |
| Range | 0.430 – 0.584 | 0.430 – 0.567 | 0.427 – 0.567 | 0.427 – 0.584 |

Number of litters, mean, standard deviation and range of the continuous litter variables evaluated in the analyses with respect to the ease of whelping.

|  | **Normal** | **Assisted** | **C-section** | **Total** |
| --- | --- | --- | --- | --- |
| **Weight of a litter in kg** | | | | |
| N | 459 | 115 | 93 | 667 |
| Mean | 3.79 | 3.84 | 3.81 | 3.80 |
| Std Dev | 0.90 | 0.92 | 0.96 | 0.91 |
| Range | 0.92 – 6.03 | 1.43 – 5.92 | 1.68 – 5.74 | 0.92 – 6.03 |
| **Average puppy weight in a litter in kg** | | | | |
| N | 459 | 115 | 93 | 667 |
| Mean | 0.48 | 0.50 | 0.50 | 0.49 |
| Std Dev | 0.05 | 0.05 | 0.06 | 0.06 |
| Range | 0.34 – 0.67 | 0.35 – 0.64 | 0.33 – 0.70 | 0.33 – 0.70 |
| **Standard deviation of the puppy weights in a litter in kg** | | | | |
| N | 459 | 115 | 93 | 667 |
| Mean | 0.043 | 0.045 | 0.049 | 0.044 |
| Std Dev | 0.023 | 0.024 | 0.029 | 0.024 |
| Range | 0.003 – 0.171 | 0.010 – 0.209 | 0.016 – 0.152 | 0.003 – 0.209 |
| **Variance of the puppy weights in a litter in kg** | | | | |
| N | 459 | 115 | 93 | 667 |
| Mean | 0.0024 | 0.0026 | 0.0033 | 0.0025 |
| Std Dev | 0.0033 | 0.0045 | 0.0045 | 0.0037 |
| Range | 0.0000 – 0.0294 | 0.0001 – 0.0437 | 0.0002 – 0.0231 | 0.0000 – 0.0437 |
| **Weight of the heaviest puppy in a litter in kg** | | | | |
| N | 459 | 115 | 93 | 667 |
| Mean | 0.53 | 0.56 | 0.57 | 0.54 |
| Std Dev | 0.06 | 0.05 | 0.07 | 0.06 |
| Range | 0.11 – 0.71 | 0.44 – 0.68 | 0.41 – 0.77 | 0.11 – 0.77 |

Comparison of the means of the adult weights (kg) of the dam.

| Parity | N | Mean | Standard Error | 95% Confidence Interval | |
| --- | --- | --- | --- | --- | --- |
| 1 | 251 | 28.07 | 0.15 | 27.78 | 28.36 |
| 2 | 186 | 28.15 | 0.17 | 27.83 | 28.48 |
| 3 | 118 | 28.39 | 0.21 | 27.98 | 28.79 |
| 4 | 73 | 28.50 | 0.23 | 28.05 | 28.95 |
| 5 | 28 | 28.50 | 0.43 | 27.66 | 29.34 |
| 6 | 11 | 28.49 | 0.61 | 27.29 | 29.69 |

One-way ANOVA clearly shows that none of the differences between the means is significant (F = 0.72, P > F = 0.61).

-----------------------------------------------------------------------------------------------------------------

Frequency of litters with respect to the quality of contractions and the number of malpositioned fetuses.

|  | Uterine contractions | |  |
| --- | --- | --- | --- |
| Number of malpositioned fetuses | normal | poor | Total |
| 0 | 506 | 14 | 520 |
| 1 | 93 | 3 | 96 |
| 2 | 25 | 3 | 28 |
| 3 or more | 21 | 2 | 23 |
| Total | 645 | 22 | 667 |

-----------------------------------------------------------------------------------------------------------------

-----------------------------------------------------------------------------------------------------------------

Comparison of the mean weight of first parity dams with the mean weight of second and more parity dams.

Two sample t test with unequal variances

| Parity | N | Mean | Std Err | Std Dev | 95% Conf Interval | |
| --- | --- | --- | --- | --- | --- | --- |
| 1 | 251 | 28.07 | 0.15 | 2.34 | 27.78 | 28.36 |
| 2 or more | 416 | 28.31 | 0.11 | 2.21 | 28.10 | 28.53 |
| combined | 667 | 28.22 | 0.09 | 2.26 | 28.05 | 28.39 |
| difference |  | -0.24 | 0.18 |  | -0.60 | 0.12 |

t = -1.32, Satterthwaite’s df = 502.47

| Hypothesis | P-value |
| --- | --- |
| mean(parity 1) – mean(parity 2 or more) = 0 |  |
| mean(parity 1) – mean(parity 2 or more) < 0 | 0.09 |
| mean(parity 1) – mean(parity 2 or more) ≠ 0 | 0.19 |
| mean(parity 1) – mean(parity 2 or more) > 0 | 0.91 |

-----------------------------------------------------------------------------------------------------------------

Comparison of the mean weight of second and more parity dams with and without an unplanned c-section.

Two sample t test with unequal variances

| C-section | N | Mean | Std Err | Std Dev | 95% Conf Interval | |
| --- | --- | --- | --- | --- | --- | --- |
| no | 284 | 28.50 | 0.14 | 2.29 | 28.23 | 28.77 |
| yes | 50 | 27.91 | 0.29 | 2.08 | 27.31 | 28.50 |
| combined | 334 | 28.41 | 0.12 | 2.27 | 28.17 | 28.66 |
| difference |  | 0.60 | 0.32 |  | -0.05 | 1.24 |

t = 1.84, Satterthwaite’s df = 71.53

| Hypothesis | P-value |
| --- | --- |
| mean(no c-section) – mean(c-section) = 0 |  |
| mean(no c-section) – mean(c-section) < 0 | 0.96 |
| mean(no c-section) – mean(c-section) ≠ 0 | 0.07 |
| mean(no c-section) – mean(c-section) > 0 | 0.04 |

-----------------------------------------------------------------------------------------------------------------

Comparison of the mean weight of first parity dams with and without an unplanned c-section.

Two sample t test with unequal variances

| C-section | N | Mean | Std Err | Std Dev | 95% Conf Interval | |
| --- | --- | --- | --- | --- | --- | --- |
| no | 175 | 28.45 | 0.18 | 2.34 | 28.10 | 28.80 |
| yes | 43 | 26.98 | 0.30 | 2.00 | 26.37 | 27.60 |
| combined | 218 | 28.16 | 0.16 | 2.34 | 27.85 | 28.47 |
| difference |  | 1.46 | 0.35 |  | 0.76 | 2.16 |

t = 4.16, Satterthwaite’s df = 73.14

| Hypothesis | P-value |
| --- | --- |
| mean(no c-section) – mean(c-section) = 0 |  |
| mean(no c-section) – mean(c-section) < 0 | 1.00 |
| mean(no c-section) – mean(c-section) ≠ 0 | 0.00 |
| mean(no c-section) – mean(c-section) > 0 | 0.00 |

-----------------------------------------------------------------------------------------------------------------
