# Supplementary material for: Factors contributing to the decision to perform a cesarean section in Labrador retrievers
Source: BMC Vet Res. 2018 Feb 27;14:57. doi: 10.1186/s12917-018-1381-8 (PMC5828337; doi:10.1186/s12917-018-1381-8)
Supplement: Supplementary file 3 — Displaying for Figs. 1, 2, 3, 4, 5 and 6 the predicted probabilities for the EOW outcomes, together with the P-values for the tests of predicted probabilities being different from zero. (DOCX 31 kb) [file 12917_2018_1381_MOESM3_ESM.docx]

**Additional file 3: Predictions for the ease of whelping and their probabilities for Figure 1 to 6**

**Figure 1**

shows predictions and their probabilities for the outcomes of the ease of whelping for the average weight of a dam and for weights differing one or two standard deviations from the average (dam kg), where no fetus was malpositioned and contractions were normal.

a) Weight of the heaviest puppy in a litter is 0.42 kg

|  | Predicted probability | | | Probability of prediction = 0 | | |
| --- | --- | --- | --- | --- | --- | --- |
| Dam kg | normal | assisted | c-section | normal | assisted | c-section |
| 23.6 | 0.860 | 0.079 | 0.061 | 0.000 | 0.000 | 0.001 |
| 25.9 | 0.895 | 0.060 | 0.045 | 0.000 | 0.000 | 0.000 |
| 28.2 | 0.922 | 0.046 | 0.033 | 0.000 | 0.000 | 0.000 |
| 30.5 | 0.942 | 0.034 | 0.024 | 0.000 | 0.001 | 0.002 |
| 32.8 | 0.957 | 0.025 | 0.017 | 0.000 | 0.005 | 0.009 |

Under the given fixed predictors, a light dam (23.6 kg) has a predicted probability of 0.061 probability points to have a c-section. This predicted probability differs significantly from zero (P < 0.001). The difference in the predicted probability of a c-section between a light dam (23.6 kg) and a heavy dam (32.8 kg) is 0.061 – 0.017 = 0.044 that is 0.04 probability points.

b) Weight of the heaviest puppy in a litter is 0.54 kg

|  | Predicted probability | | | Probability of prediction = 0 | | |
| --- | --- | --- | --- | --- | --- | --- |
| Dam kg | normal | assisted | c-section | normal | assisted | c-section |
| 23.6 | 0.737 | 0.138 | 0.125 | 0.000 | 0.000 | 0.000 |
| 25.9 | 0.794 | 0.112 | 0.094 | 0.000 | 0.000 | 0.000 |
| 28.2 | 0.842 | 0.088 | 0.070 | 0.000 | 0.000 | 0.000 |
| 30.5 | 0.881 | 0.068 | 0.051 | 0.000 | 0.000 | 0.000 |
| 32.8 | 0.911 | 0.052 | 0.038 | 0.000 | 0.000 | 0.000 |

c) Weight of the heaviest puppy in a litter is 0.66 kg

|  | Predicted probability | | | Probability of prediction = 0 | | |
| --- | --- | --- | --- | --- | --- | --- |
| Dam kg | normal | assisted | c-section | normal | assisted | c-section |
| 23.6 | 0.560 | 0.201 | 0.239 | 0.000 | 0.000 | 0.000 |
| 25.9 | 0.637 | 0.178 | 0.185 | 0.000 | 0.000 | 0.000 |
| 28.2 | 0.708 | 0.151 | 0.141 | 0.000 | 0.000 | 0.000 |
| 30.5 | 0.770 | 0.123 | 0.106 | 0.000 | 0.000 | 0.000 |
| 32.8 | 0.822 | 0.098 | 0.079 | 0.000 | 0.000 | 0.001 |

The difference in the predicted probability of a c-section between a light dam (23.6 kg) and a heavy dam (32.8 kg) is 0.239 – 0.079 = 0.160 that is 0.16 probability points.

**Figure 2**

shows predictions and their probabilities for the outcomes of the ease of whelping for the average weight of a dam and for weights differing one or two standard deviations from the average (dam kg), where no fetus was malpositioned and contractions were poor.

a) Weight of the heaviest puppy in a litter is 0.42 kg

|  | Predicted probability | | | Probability of prediction = 0 | | |
| --- | --- | --- | --- | --- | --- | --- |
| Dam kg | normal | assisted | c-section | normal | assisted | c-section |
| 23.6 | 0.277 | 0.213 | 0.511 | 0.022 | 0.000 | 0.001 |
| 25.9 | 0.345 | 0.224 | 0.431 | 0.011 | 0.000 | 0.003 |
| 28.2 | 0.422 | 0.225 | 0.354 | 0.005 | 0.000 | 0.011 |
| 30.5 | 0.502 | 0.214 | 0.284 | 0.002 | 0.000 | 0.030 |
| 32.8 | 0.582 | 0.195 | 0.223 | 0.001 | 0.000 | 0.063 |

The difference in the predicted probability of a c-section between a light dam (23.6 kg) and a heavy dam (32.8 kg) is 0.511 – 0.223 = 0.288 that is 0.29 probability points.

b) Weight of the heaviest puppy in a litter is 0.54 kg

|  | Predicted probability | | | Probability of prediction = 0 | | |
| --- | --- | --- | --- | --- | --- | --- |
| Dam kg | normal | assisted | c-section | normal | assisted | c-section |
| 23.6 | 0.148 | 0.155 | 0.697 | 0.033 | 0.002 | 0.000 |
| 25.9 | 0.193 | 0.182 | 0.625 | 0.021 | 0.000 | 0.000 |
| 28.2 | 0.249 | 0.205 | 0.547 | 0.014 | 0.000 | 0.000 |
| 30.5 | 0.314 | 0.220 | 0.466 | 0.011 | 0.000 | 0.001 |
| 32.8 | 0.387 | 0.226 | 0.387 | 0.008 | 0.000 | 0.008 |

The difference in the predicted probability of a c-section between a light dam (23.6 kg) and a heavy dam (32.8 kg) is 0.697 – 0.387 = 0.310 that is 0.31 probability points.

c) Weight of the heaviest puppy in a litter is 0.66 kg

|  | Predicted probability | | | Probability of prediction = 0 | | |
| --- | --- | --- | --- | --- | --- | --- |
| Dam kg | normal | assisted | c-section | normal | assisted | c-section |
| 23.6 | 0.073 | 0.092 | 0.835 | 0.060 | 0.025 | 0.000 |
| 25.9 | 0.098 | 0.116 | 0.786 | 0.044 | 0.011 | 0.000 |
| 28.2 | 0.131 | 0.143 | 0.726 | 0.037 | 0.004 | 0.000 |
| 30.5 | 0.172 | 0.170 | 0.658 | 0.035 | 0.001 | 0.000 |
| 32.8 | 0.223 | 0.195 | 0.582 | 0.036 | 0.000 | 0.000 |

The difference in the predicted probability of a c-section between a light dam (23.6 kg) and a heavy dam (32.8 kg) is 0.835 – 0.582 = 0.253 that is 0.25 probability points.

The differences in the predicted probability of a c-section between a light heaviest puppy (0.42 kg) and a heavy heaviest puppy (0.66 kg) over the range of the weight of the dam (23.6 kg to 32.8 kg) are 0.32, 0.36, 0.37, 0.37 and 036 probability points.

The differences in the predicted probability of an assisted whelping between Figure 1 and Figure 2 where the weight of the dam is 28.2 kg and the weight of the heaviest puppy in the litter 0.54 kg is 0.205 – 0.088 = 0.117 that is 0.12 probability points.

**Figure 3**

shows predictions and their probabilities for the outcomes of the ease of whelping for the average weight of a dam and for weights differing one or two standard deviations from the average (dam kg), where one fetus was malpositioned and contractions were normal.

a) Weight of the heaviest puppy in a litter is 0.42 kg

|  | Predicted probability | | | Probability of prediction = 0 | | |
| --- | --- | --- | --- | --- | --- | --- |
| Dam kg | normal | assisted | c-section | normal | assisted | c-section |
| 23.6 | 0.382 | 0.441 | 0.177 | 0.000 | 0.000 | 0.000 |
| 25.9 | 0.460 | 0.405 | 0.135 | 0.000 | 0.000 | 0.000 |
| 28.2 | 0.541 | 0.358 | 0.102 | 0.000 | 0.000 | 0.000 |
| 30.5 | 0.619 | 0.305 | 0.076 | 0.000 | 0.000 | 0.003 |
| 32.8 | 0.692 | 0.252 | 0.056 | 0.000 | 0.000 | 0.014 |

b) Weight of the heaviest puppy in a litter is 0.54 kg

|  | Predicted probability | | | Probability of prediction = 0 | | |
| --- | --- | --- | --- | --- | --- | --- |
| Dam kg | normal | assisted | c-section | normal | assisted | c-section |
| 23.6 | 0.219 | 0.459 | 0.322 | 0.000 | 0.000 | 0.000 |
| 25.9 | 0.279 | 0.465 | 0.256 | 0.000 | 0.000 | 0.000 |
| 28.2 | 0.348 | 0.452 | 0.199 | 0.000 | 0.000 | 0.000 |
| 30.5 | 0.425 | 0.423 | 0.153 | 0.000 | 0.000 | 0.000 |
| 32.8 | 0.505 | 0.380 | 0.115 | 0.000 | 0.000 | 0.001 |

The differences in the predicted probability of an assisted whelping of Figure 3 compared to Figure 1 and Figure 2 where the weight of the dam is 28.2 kg and the weight of the heaviest puppy in the litter 0.54 kg is 0.452 – 0.088 = 0.364 that is 0.36 probability points and 0.452 – 0.205 = 0.247 that is 0.25 probability points, respectively.

The differences in the predicted probability of a c-section of Figure 3 compared to Figure 1 and Figure 2 where the weight of the dam is 28.2 kg and the weight of the heaviest puppy in the litter 0.54 kg is 0.199 – 0.070 = 0.129 that is 0.13 probability points and 0.199 – 0.547 = -0.348 that is -0.35 probability points, respectively.

c) Weight of the heaviest puppy in a litter is 0.66 kg

|  | Predicted probability | | | Probability of prediction = 0 | | |
| --- | --- | --- | --- | --- | --- | --- |
| dam kg | normal | assisted | c-section | normal | assisted | c-section |
| 23.6 | 0.113 | 0.376 | 0.511 | 0.002 | 0.000 | 0.000 |
| 25.9 | 0.150 | 0.420 | 0.431 | 0.000 | 0.000 | 0.000 |
| 28.2 | 0.195 | 0.451 | 0.354 | 0.000 | 0.000 | 0.000 |
| 30.5 | 0.251 | 0.465 | 0.284 | 0.000 | 0.000 | 0.000 |
| 32.8 | 0.316 | 0.460 | 0.223 | 0.000 | 0.000 | 0.000 |

**Figure 4**

shows predictions and their probabilities for the outcomes of the ease of whelping for the average weight of a dam and for weights differing one or two standard deviations from the average (dam kg), where one fetus was malpositioned and contractions were poor.

a) Weight of the heaviest puppy in a litter is 0.42 kg

|  | Predicted probability | | | Probability of prediction = 0 | | |
| --- | --- | --- | --- | --- | --- | --- |
| Dam kg | normal | assisted | c-section | normal | assisted | c-section |
| 23.6 | 0.037 | 0.187 | 0.777 | 0.105 | 0.036 | 0.000 |
| 25.9 | 0.050 | 0.234 | 0.716 | 0.098 | 0.020 | 0.000 |
| 28.2 | 0.068 | 0.286 | 0.646 | 0.100 | 0.010 | 0.000 |
| 30.5 | 0.092 | 0.339 | 0.569 | 0.107 | 0.003 | 0.001 |
| 32.8 | 0.122 | 0.389 | 0.489 | 0.119 | 0.000 | 0.008 |

b) Weight of the heaviest puppy in a litter is 0.54 kg

|  | Predicted probability | | | Probability of prediction = 0 | | |
| --- | --- | --- | --- | --- | --- | --- |
| Dam kg | normal | assisted | c-section | normal | assisted | c-section |
| 23.6 | 0.017 | 0.098 | 0.884 | 0.083 | 0.049 | 0.000 |
| 25.9 | 0.023 | 0.129 | 0.847 | 0.075 | 0.035 | 0.000 |
| 28.2 | 0.032 | 0.167 | 0.801 | 0.077 | 0.028 | 0.000 |
| 30.5 | 0.044 | 0.212 | 0.744 | 0.088 | 0.022 | 0.000 |
| 32.8 | 0.059 | 0.263 | 0.678 | 0.107 | 0.017 | 0.000 |

c) Weight of the heaviest puppy in a litter is 0.66 kg

|  | Predicted probability | | | Probability of prediction = 0 | | |
| --- | --- | --- | --- | --- | --- | --- |
| Dam kg | normal | assisted | c-section | normal | assisted | c-section |
| 23.6 | 0.008 | 0.048 | 0.944 | 0.096 | 0.074 | 0.000 |
| 25.9 | 0.011 | 0.065 | 0.924 | 0.085 | 0.060 | 0.000 |
| 28.2 | 0.015 | 0.087 | 0.898 | 0.085 | 0.054 | 0.000 |
| 30.5 | 0.020 | 0.115 | 0.865 | 0.094 | 0.053 | 0.000 |
| 32.8 | 0.028 | 0.150 | 0.822 | 0.113 | 0.055 | 0.000 |

**Figure 5**

shows predictions and their probabilities for the outcomes of the ease of whelping for the number of malpositioned fetuses (NMF), where contractions were normal and the heaviest puppy of a litter was average with 0.54 kg.

a) Weight of the dam is 23.6 kg

|  | Predicted probability | | | Probability of prediction = 0 | | |
| --- | --- | --- | --- | --- | --- | --- |
| NMF | normal | assisted | c-section | normal | assisted | c-section |
| 0 | 0.737 | 0.138 | 0.125 | 0.000 | 0.000 | 0.000 |
| 1 | 0.219 | 0.459 | 0.322 | 0.000 | 0.000 | 0.000 |
| 2 | 0.047 | 0.479 | 0.474 | 0.013 | 0.000 | 0.000 |
| 3 | 0.015 | 0.491 | 0.494 | 0.194 | 0.000 | 0.000 |

The increase of the predicted probability for a c-section from 0 to 2 malpositioned fetuses is 0.474 – 0.125 = 0.349 that is 0.35 probability points.

The increase of the predicted probability for a c-section from 2 to 3 malpositioned fetuses is 0.494 – 0.474 = 0.020 that is 0.02 probability points.

The increase of the predicted probability for an assisted delivery from 0 to 1 malpositioned fetuses is 0.459 – 0.138 = 0.321 that is 0.32 probability points.

The increase of the predicted probability for an assisted delivery from 1 to 3 malpositioned fetuses is 0.491 – 0.459 = 0.032 that is 0.03 probability points.

b) Weight of the dam is 28.2 kg

|  | Predicted probability | | | Probability of prediction = 0 | | |
| --- | --- | --- | --- | --- | --- | --- |
| NMP | normal | assisted | c-section | normal | assisted | c-section |
| 0 | 0.842 | 0.088 | 0.070 | 0.000 | 0.000 | 0.000 |
| 1 | 0.348 | 0.452 | 0.199 | 0.000 | 0.000 | 0.000 |
| 2 | 0.086 | 0.593 | 0.321 | 0.003 | 0.000 | 0.000 |
| 3 | 0.028 | 0.634 | 0.338 | 0.171 | 0.000 | 0.000 |

The increase of the predicted probability for an assisted delivery from 0 to 2 malpositioned fetuses is 0.593 – 0.0.088 = 0.505 that is 0.51 probability points.

The increase of the predicted probability for an assisted delivery from 2 to 3 or more malpositioned fetuses is 0.634 – 0.593 = 0.041 that is 0.04 probability points.

c) Weight of the dam is 32.8 kg

|  | Predicted probability | | | Probability of prediction = 0 | | |
| --- | --- | --- | --- | --- | --- | --- |
| NMP | normal | assisted | c-section | normal | assisted | c-section |
| 0 | 0.911 | 0.052 | 0.038 | 0.000 | 0.000 | 0.000 |
| 1 | 0.505 | 0.380 | 0.115 | 0.000 | 0.000 | 0.001 |
| 2 | 0.152 | 0.650 | 0.199 | 0.007 | 0.000 | 0.000 |
| 3 | 0.052 | 0.736 | 0.212 | 0.178 | 0.000 | 0.009 |

The increase of the predicted probability for a c-section from 0 to 2 malpositioned fetuses is 0.199 – 0.0.038 = 0.161 that is 0.16 probability points.

The increase of the predicted probability for a c-section from 2 to 3 malpositioned fetuses is 0.212 – 0.199 = 0.013 that is 0.01 probability points.

The increase of the predicted probability for an assisted delivery from 0 to 2 malpositioned fetuses is 0.650 – 0.0.052 = 0.598 that is 0.60 probability points.

The increase of the predicted probability for an assisted delivery from 2 to 3 malpositioned fetuses is 0.736 – 0.650 = 0.086 that is 0.09 probability points.

**Figure 6**

shows predictions and their probabilities for the outcomes of the ease of whelping for the number of malpositioned fetuses (NMF), where contractions were poor and the heaviest puppy of a litter was average with 0.54 kg.

a) Weight of the dam is 23.6 kg

|  | Predicted probability | | | Probability of prediction = 0 | | |
| --- | --- | --- | --- | --- | --- | --- |
| NMF | normal | assisted | c-section | normal | assisted | c-section |
| 0 | 0.148 | 0.155 | 0.697 | 0.033 | 0.002 | 0.000 |
| 1 | 0.017 | 0.098 | 0.884 | 0.083 | 0.049 | 0.000 |
| 2 | 0.003 | 0.061 | 0.936 | 0.132 | 0.074 | 0.000 |
| 3 | 0.001 | 0.059 | 0.940 | 0.281 | 0.120 | 0.000 |

b) Weight of the dam is 28.2 kg

|  | Predicted probability | | | Probability of prediction = 0 | | |
| --- | --- | --- | --- | --- | --- | --- |
| NMP | normal | assisted | c-section | normal | assisted | c-section |
| 0 | 0.249 | 0.205 | 0.547 | 0.014 | 0.000 | 0.000 |
| 1 | 0.032 | 0.167 | 0.801 | 0.077 | 0.028 | 0.000 |
| 2 | 0.006 | 0.110 | 0.884 | 0.129 | 0.060 | 0.000 |
| 3 | 0.002 | 0.106 | 0.892 | 0.277 | 0.102 | 0.000 |

c) Weight of the dam is 32.8 kg

|  | Predicted probability | | | Probability of prediction = 0 | | |
| --- | --- | --- | --- | --- | --- | --- |
| NMP | normal | assisted | c-section | normal | assisted | c-section |
| 0 | 0.387 | 0.226 | 0.387 | 0.008 | 0.000 | 0.008 |
| 1 | 0.059 | 0.263 | 0.678 | 0.107 | 0.017 | 0.000 |
| 2 | 0.011 | 0.189 | 0.800 | 0.165 | 0.065 | 0.000 |
| 3 | 0.003 | 0.184 | 0.812 | 0.299 | 0.102 | 0.000 |
